# Optimization by Adaptive Stochastic Descent

**DOI:** 10.1371/journal.pone.0192944

**Published:** 2018-03-16

**Authors:** Cliff C. Kerr, Salvador Dura-Bernal, Tomasz G. Smolinski, George L. Chadderdon, David P. Wilson

**Affiliations:** 1 Complex Systems Group, School of Physics, University of Sydney, Sydney, NSW, Australia; 2 Optima Consortium for Decision Science, Melbourne, VIC, Australia; 3 Centre for Population Health, Burnet Institute, Melbourne, VIC, Australia; 4 Department of Physiology and Pharmacology, SUNY Downstate Medical Center, Brooklyn, NY, United States of America; 5 Department of Computer and Information Sciences, Delaware State University, Dover, DE, United States of America; Universitatsmedizin Greifswald, GERMANY

## Abstract

When standard optimization methods fail to find a satisfactory solution for a parameter fitting problem, a tempting recourse is to adjust parameters manually. While tedious, this approach can be surprisingly powerful in terms of achieving optimal or near-optimal solutions. This paper outlines an optimization algorithm, Adaptive Stochastic Descent (ASD), that has been designed to replicate the essential aspects of manual parameter fitting in an automated way. Specifically, ASD uses simple principles to form probabilistic assumptions about (a) which parameters have the greatest effect on the objective function, and (b) optimal step sizes for each parameter. We show that for a certain class of optimization problems (namely, those with a moderate to large number of scalar parameter dimensions, especially if some dimensions are more important than others), ASD is capable of minimizing the objective function with far fewer function evaluations than classic optimization methods, such as the Nelder-Mead nonlinear simplex, Levenberg-Marquardt gradient descent, simulated annealing, and genetic algorithms. As a case study, we show that ASD outperforms standard algorithms when used to determine how resources should be allocated in order to minimize new HIV infections in Swaziland.

## Introduction

Consider a human H who is attempting to minimize a nonlinear objective function, *E* = *f*(**x**), by manually adjusting parameters in the vector **x**. H typically begins with a uniform prior regarding which parameters to vary, and chooses step sizes that are a fixed fraction (*e.g.*, 10%) of the initial parameter values. H will then pseudorandomly choose one or more parameters to adjust. Every time a parameter *x*_*i*_ is found to reduce *E*, the probability that H will select *x*_*i*_ in the future increases; conversely, if changes in *x*_*i*_ are not found to improve *E*, the probability that H will select *x*_*i*_ decreases (formally, H forms “hunches” about which parameters are “good”). H also adaptively adjusts the step size based on the information H obtains about the curvature of parameter space with respect to each dimension (*e.g.*, if Δ*E*/Δ*x*_*i*_ ≈ const. over multiple iterations, H will increase the step size). Despite its drawbacks, the adaptive nature of manual parameter fitting makes it a remarkably powerful method.

Thus, despite the smörgåsbord of available automated optimization algorithms, manual fitting of parameters remains a familiar bane of researchers (*e.g.*, [1, 2]), especially in cases where evaluations of the objective function are computationally intensive, such as climate models [3], neuronal network models [4–6], or detailed epidemiological models [7]. However, it is difficult to estimate how commonly manual parameter fitting is performed, since authors often do not explicitly mention its use (*e.g.*, [8]).

In many types of optimization problems, it is more important to need only a small number of function evaluations to find a reasonable local minimum than it is to find the global minimum [9, 10]. Indeed, the latter may be ill-defined given the large uncertainties that are often present when models of complex systems are fitted to empirical data, as in the citations listed above.

With the increasing availability of high-performance computers and clusters [11], easily parallelizable optimization methods such as evolutionary algorithms (where different individuals can be run on different cores) and Monte Carlo methods (where different initializations can be run on different cores) have a notable advantage for certain types of problems. The common theme in these algorithms is the ability to use a different random seed for each parallel instance. However, as the size of parameter space increases, the advantage of this approach is diluted: whereas a 3- or even 5-dimensional parameter space may be reasonably densely sampled by a Monte Carlo initialization, a 20- or 100-dimensional space cannot. This is because parameter space grows exponentially with an increasing number of dimensions, whereas parallelization increases sampling rates linearly.

In high-dimensional parameter spaces, it is unlikely that all parameters contribute equally to the objective function. Identifying those that contribute more, thereby allowing computational resources to be focused on them, has the potential to significantly reduce the total number of function evaluations required. Despite humans’ limited capacity to implement Bayesian-optimal strategies [12, 13], we speculate that this adaptive approach to both parameter selection and step size is the key reason why manual parameter fitting can be highly effective.

The aim of this paper is to present a random search algorithm, Adaptive Stochastic Descent (ASD), that was inspired by manual parameter fitting and is intended to be a simpler alternative to more complex optimization methods. ASD is most applicable to optimization problems with more than approximately 5 dimensions—*i.e.*, large enough so that performing function evaluations across all dimensions is inefficient. ASD forms the core of the optimization algorithm used in the Optima suite of tools (optimamodel.com), most notably Optima HIV [14], and as such has already been extensively used and validated for calibrating epidemic models and determining optimal resource allocations [15–22]. The algorithm has also been applied to fitting a spiking neuronal network model to electrophysiology data from individual rat brains [23], and has been used in ongoing work calibrating a neural field model to reproduces impulse responses in sleep EEG data [24]. Here we also compare ASD to traditional algorithms using two classic optimization test problems, and provide an extended case study on optimally allocating resources for HIV interventions using a detailed model of Swaziland’s HIV epidemic.

ASD is provided under the open-source MIT License. Python and MATLAB versions are available for download from thekerrlab.com/asd or via GitHub at github.com/thekerrlab/asd.

## Basic algorithm

Consider an objective function *E* = *f*(**x**), where *E* is the scalar error (or other quantity) to be minimized (or maximized) and **x** = [*x*_1_, *x*_2_, …, *x*_*n*_] is an *n*-element vector of parameters. There are 2*n* possible directions *j* to step in: an increase or decrease in the value of each parameter. Associated with each parameter *x*_*i*_ are (a) two initial step sizes: $s_{j}=s_{i}^{+}$ or $s_{i}^{-}$, which define the step size in the directions of increasing or decreasing *x*_*i*_, respectively (*i.e.*, $s_{i}^{+}>0$ and $s_{i}^{-}<0$); and (b) two initial probabilities: $p_{j}=p_{i}^{+}$ or $p_{i}^{-}$, which define the likelihood of selecting direction *j* (for a uniform prior, *p*_*j*_ = 1/2*n*—satisfying the requirement that $\sum p=\sum_{j=1}^{2n}p_{j}=1$). Thus, the vectors **s** and **p** have length 2*n*.

At each step *k*, the algorithm maps a random variable *α* ∈ (0, 1) onto **p**, thereby choosing a direction *j* ∈ (1…2*n*) and a corresponding parameter *i* = ⌈*j*/2⌉ ∈ (1…*n*), where ⌈·⌉ denotes the ceiling operator. The algorithm then evaluates

$$\begin{array}{c} E_{k}^{\pm }=f\left(x+\delta \left(i\right)\right), \end{array}$$ (1)

where *δ*(*i*) is an *n*-element vector such that *δ*_*i*_ = *s*_*j*_ and 0 otherwise. Then:

1. If $E_{k}^{\pm }<E_{k-1}$:
  - The new parameter value is adopted: *x*_*i*_ → *x*_*i*_ + *s*_*j*_;
  - The error is updated: $E_{k}\to E_{k}^{\pm }$;
  - *s*_*j*_ is increased: *s*_*j*_ → *s*_*j*_ ⋅ *s*_*inc*_ (where *s*_*inc*_ > 1);
  - *p*_*j*_ is increased: *p*_*j*_ → *p*_*j*_ ⋅ *p*_*inc*_ (where *p*_*inc*_ > 1), and **p** is renormalized such that ∑**p** = 1.
2. Otherwise:
  - The parameter vector **x** and error *E* are not changed;
  - *s*_*j*_ is decreased: *s*_*j*_ → *s*_*j*_/*s*_*dec*_ (where *s*_*dec*_ > 1);
  - *p*_*j*_ is decreased: *p*_*j*_ → *p*_*j*_/*p*_*dec*_ (where *p*_*dec*_ > 1), and **p** is renormalized as above.

The algorithm thus has four metaparameters: *s*_*inc*_, *s*_*dec*_, *p*_*inc*_, and *p*_*dec*_. In general, the smoother and more linear the objective function is, the larger the learning rates should be; the choice of *s*_*inc*_ = *s*_*dec*_ = *p*_*inc*_ = *p*_*dec*_ = 2 has been found to work well for both simple test cases as well as optimizing complex epidemiological models, although values from approximately 1.2 to 3 were found to have broadly similar performance. In addition to these metaparameters, three initial value vectors need to be specified: the initial parameter vector **x**_**0**_, step sizes **s** (which in general can be initialized as a fixed fraction of the corresponding initial parameter value, *e.g.* 20%, unless the initial value is zero), and probabilities **p** (where typically *p*_*j*_ = 1/2*n* suffices for an *n*-parameter problem).

By modifying **s** and **p** after each iteration, the algorithm learns which directions are most effective to step in and by how much (in the sense that it updates its choices of **s** and **p** by their initial states depending on accumulated evidence). This, combined with the stochastic choice of which parameters to modify on each iteration, resembles the way in which humans (imperfectly) perform Bayesian decision-making in situations such as *N*-armed bandit problems [13].

The criteria for terminating the algorithm can be specified in the same way as for traditional optimization algorithms. The most common choices for termination are when changes in parameter values (*i.e.*, Δ*x*) and/or improvements in the objective function (*i.e.*, Δ*E*) are below a given absolute or relative threshold (*e.g.*, 10^−6^) for a given number of iterations (*e.g.*, 50).

## Extensions to the algorithm

This section describes several modifications to the basic algorithm that may make it more suitable for a broader range of optimization problems.

To circumvent the problem of local minima, the method may be used with Monte Carlo initialization [25]. In this case, the ASD algorithm is repeated multiple times (typically, 10^1^ − 10^3^) with pseudorandom choices of **x**_**0**_. The use of multiple starting points helps achieve the balance between “exploration and exploitation” (exploring the entire feasible region of parameter space versus exploring the most promising subregions), which is critical for efficient global search [26]. This is the approach used in Optima HIV, where typically up to 10 Monte Carlo initializations are used. When we applied ASD to each of the 54 different Optima HIV models that correspond to the countries comprising 80% of the global burden of HIV [27], we found that a single initialization converged on the global optimum for 38 (70%) of the models, while 10 initializations converged on the global optimum for all but one model (98%).

Another approach for circumventing the problem of local minima is a probabilistic step acceptance process, similar to that used in simulated annealing or the Metropolis-Hastings algorithm [28]. Here, instead of always performing step 2 of the algorithm if the new iteration does not reduce error, step 1 is performed with nonzero acceptance ratio *ρ*, where *ρ* is a function of the change in error; *e.g.*, $\rho \propto E_{k-1}/E_{k}^{\pm }$. Although the parameter set resulting from each iteration can be kept, as in a Metropolis-Hastings algorithm, the value of doing so is limited since the asymptotic distribution of parameter sets is not guaranteed to reach a stationary distribution, due to the adaptive method for choosing which parameters to vary. Instead, it would suffice to keep two parameter sets, the current one and the best one. As a simpler alternative to implementing a Metropolis-Hastings approach, rather than always reducing the step size if the new iteration does not reduce the error, the step size could have a nonzero probability of increasing, potentially allowing the algorithm to escape local minima.

Note that in the limit of infinite iterations, the basic ASD algorithm will not almost surely converge to the global optimum, since the step size will asymptotically converge to zero if the algorithm is in a location of parameter space such that its step size in all dimensions is smaller than the size of the local minimum’s basin of attraction. However, the algorithm will almost surely converge to the global optimum if probabilistic step acceptance is implemented (or if step sizes have nonzero probability of increasing when an evaluation does not result in improvement). Formally, multiple initializations do not suffice to almost surely converge unless they are infinite in number. However, in practice, depending on the smoothness and monotonicity of the objective function, multiple initializations typically allow the exploration of global parameter space (and thus convergence) more efficiently than probabilistic step acceptance.

In some cases it may be desirable to allow assumptions about the scale or relative importance of parameters to be incorporated, in which case the assumptions of uniform priors **p** and uniform initial step sizes **s** can easily be relaxed. However, due to the adaptive nature of the algorithm, even silly initial choices of **p** and **s** will be corrected, as long as all *p*_*j*_ and *s*_*j*_ are nonzero. In general, choices of *s*_*j*_ or *p*_*j*_ that are too small are more problematic than ones that too large, since the latter will be corrected with each iteration that fails to improve the objective function.

To incorporate additional information about the change in the objective function, rather than updating the probability *p*_*j*_ by a fixed amount after each successful iteration, the change in *p*_*j*_ (Δ*p*_*j*_) can be a function of the change in the objective function *E* (Δ*E*), such that a larger Δ*E* results in a larger Δ*p*_*j*_, as in simultaneous perturbation stochastic approximation [29]. However, since the expected change in *E* at step *k* is proportional to both |*E*_*k*_ − min(*E*)| and the ratio of the step size to the characteristic scale of each parameter, and since in general neither of these quantities are known, the constant of proportionality between Δ*p*_*j*_ and Δ*E* cannot typically be estimated *a priori*. One can partially circumvent this problem by comparing the current Δ*E* to its previous values; however, more weight would need to be given to more recent values, since Δ*E* tends to decrease as the algorithm converges on a solution.

The assumption of local linearity can be relaxed by varying multiple parameters on a single iteration. However, assuming a separate probability is stored for each parameter combination, this reduces the learning rate; for an *n*-parameter problem, modifying a single parameter at each iteration results in a learning rate of 1/2*n* on average for each parameter; in the limit where all possible combinations of parameters are considered, the learning rate would be 1/2^2*n*^. While manageable for small numbers of parameters (*e.g.*, ≤4), this quickly becomes intractable as the number of parameters grows. Conversely, if multiple parameters are modified simultaneously, the probabilities of all modified parameters could be updated simultaneously; this approach is likely to be most effective in very high-dimensional systems where the function *E* is nearly flat with respect to many of the dimensions, in which case varying parameters one by one may be time-consuming. The superior performance of simulated annealing compared to ASD for small numbers of function evaluations in the 10-parameter Rosenbrock’s valley problem discussed below is likely due to this effect.

Finally, although only loosely inspired by Bayesian principles, the ASD algorithm could potentially be adapted to implement them more rigorously. While a more formal Bayesian approach may be desirable in certain situations, in general it is difficult to determine whether new information should be used to update the existing distribution, or whether the system is in a sufficiently dissimilar part of the parameter space that information from much earlier iterations is no longer relevant. Nonetheless, for certain problems, additional capacity for adaptation may be beneficial. For example, as shown below, the basic implementation of ASD described above performs poorly in cases where the objective function is dominated by nonlinear parameter interactions, as in the classic version of Rosenbrock’s valley; for this particular problem, an algorithm that was capable of learning nonlinear parameter combinations would be far more efficient.

## Comparison to other optimization methods

Here we compare ASD to four standard optimization methods: the Nelder-Mead nonlinear simplex algorithm [30], Levenberg-Marquardt gradient descent [31], simulated annealing [32], and a genetic algorithm [33]. All methods were implemented in MATLAB 2012b (The MathWorks, Nantick, MA), via the Optimization Toolbox functions “fminsearch”, “lsqnonlin”, “simulannealbnd”, and “ga”, respectively. These algorithms are also available in the “optimize” module of the Python package SciPy via “minimize(method = ’Nelder-Mead’)”, “leastsq()”, and “anneal()”, respectively (genetic algorithms are not available in SciPy, but are available via other modules). We chose these methods to compare against since, like ASD, they have relatively simple implementations and relatively few metaparameters that need to be specified.

For ASD, we used metaparameters *s*_*inc*_ = *p*_*inc*_ = *s*_*dec*_ = *p*_*dec*_ = 2, initial step sizes *s*_*j*_ of 20% of the parameter values in **x**_**0**_ (which are given below; the step size for any parameter with an initial value of 0 is the mean of the other step sizes), and uniform initial probabilities *p*_*j*_ (*i.e.*, 1/2*n* for an *n*-dimensional problem). MATLAB’s default metaparameters were used for the other four algorithms, except that the initial temperature of the simulated annealing algorithm was set to be equal to 10 ⋅ 〈|**x**_**0**_|〉 following manual exploration of metaparameter space, since the default choice of 100 did not generalize well across problems of different scales. Indeed, one of the major disadvantages of this type of algorithm is its sensitivity to the values of its metaparameters [34].

To test this suite of algorithms, we used original and modified versions of the two classic optimization problems used for illustrating the simplex algorithm [30]:

1. Rosenbrock’s parabolic valley (two-dimensional):
  $$\begin{array}{c} E=100{\left(x_{2}-x_{1}^{2}\right)}^{2}+{\left(1-x_{1}\right)}^{2}, \end{array}$$ (2)
  with the starting point at **x** = (−1.2, 1). The optimum is at **x** = (1, 1).
2. A modified 10-dimensional version of Rosenbrock’s valley, with the functional form as given in Eq 2, but with a 10-element parameter vector **x**; the remaining 8 parameters do not contribute to the objective function. The starting point is at **x** = (1.5, −1.5, 0, 0 … 0). The optimum is at **x** = (1, 1, *ω*_1_ … *ω*_8_), where *ω*_1_ … *ω*_8_ can be any real numbers.
3. A 4-dimensional Powell’s quartic function, modified to be *N*-dimensional:
  $$\begin{array}{c} E=\sum ({\left(x_{a}+10x_{b}\right)}^{2}+5{\left(x_{c}-x_{d}\right)}^{2}+{\left(x_{b}-2x_{c}\right)}^{4}+10{\left(x_{a}-x_{d}\right)}^{4}), \end{array}$$ (3)
  where **x**_**q**_ is a vector of length *N*/4 (and note that vector operations are performed pointwise). The starting point is at $x_{a}=\left(\bar{3}\right)$, $x_{b}=\left(\bar{-1}\right)$, $x_{c}=\left(\bar{0}\right)$, and $x_{d}=\left(\bar{1}\right)$, where each component is repeated *N*/4 times. The optimum is at **x** = (**x**_**a**_, **x**_**b**_, **x**_**c**_, **x**_**d**_) = (0, 0, 0, … 0). For example, if *N* = 4 (as in the original), then **x**_**0**_ = (3, −1, 0, 1) and **x**_opt_ = (0, 0, 0, 0); if *N* = 8, then **x**_**0**_ = (3, 3, −1, −1, 0, 0, 1, 1) and **x**_opt_ = (0, 0, 0, 0, 0, 0, 0, 0). Here, we used 4, 12, 20, and 100-dimensional versions of Powell’s function.

**Fig 1:**
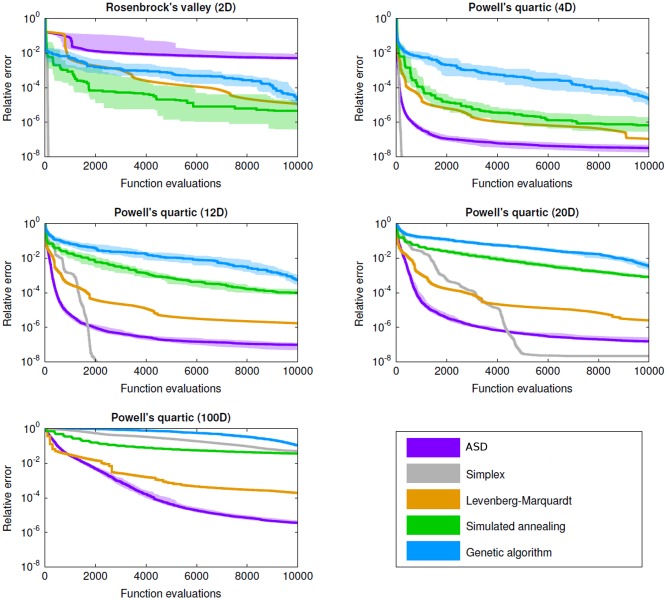
Performance of ASD compared to standard nonlinear optimization algorithms. The four algorithms used are Nelder-Mead nonlinear simplex, Levenberg-Marquardt gradient descent, simulated annealing, and genetic algorithms. The x-axis shows the number of individual function evaluations, while the y-axis shows the error relative to the starting point. Standard methods—especially the simplex method—are most efficient for low-dimensional problems (*e.g.*, Rosenbrock’s valley), in many cases ASD is the most efficient algorithm for high-dimensional parameter spaces (*e.g.*, the 100-dimensional version of Powell’s quartic function). For the stochastic methods (ASD, simulated annealing, and the genetic algorithm), the shaded regions show the interquartile range for 40 different random seeds.

The results from applying each of these algorithms to each of the three test problems is shown in Fig 1. For the stochastic algorithms (ASD, simulated annealing, and genetic algorithms), the interval shown represents the interquartile range for 40 different random seeds. For most test problems and iterations, these interquartile ranges did not overlap, suggesting that the intrinsic differences between the algorithms are more important than their stochastic components.

**Fig 2:**
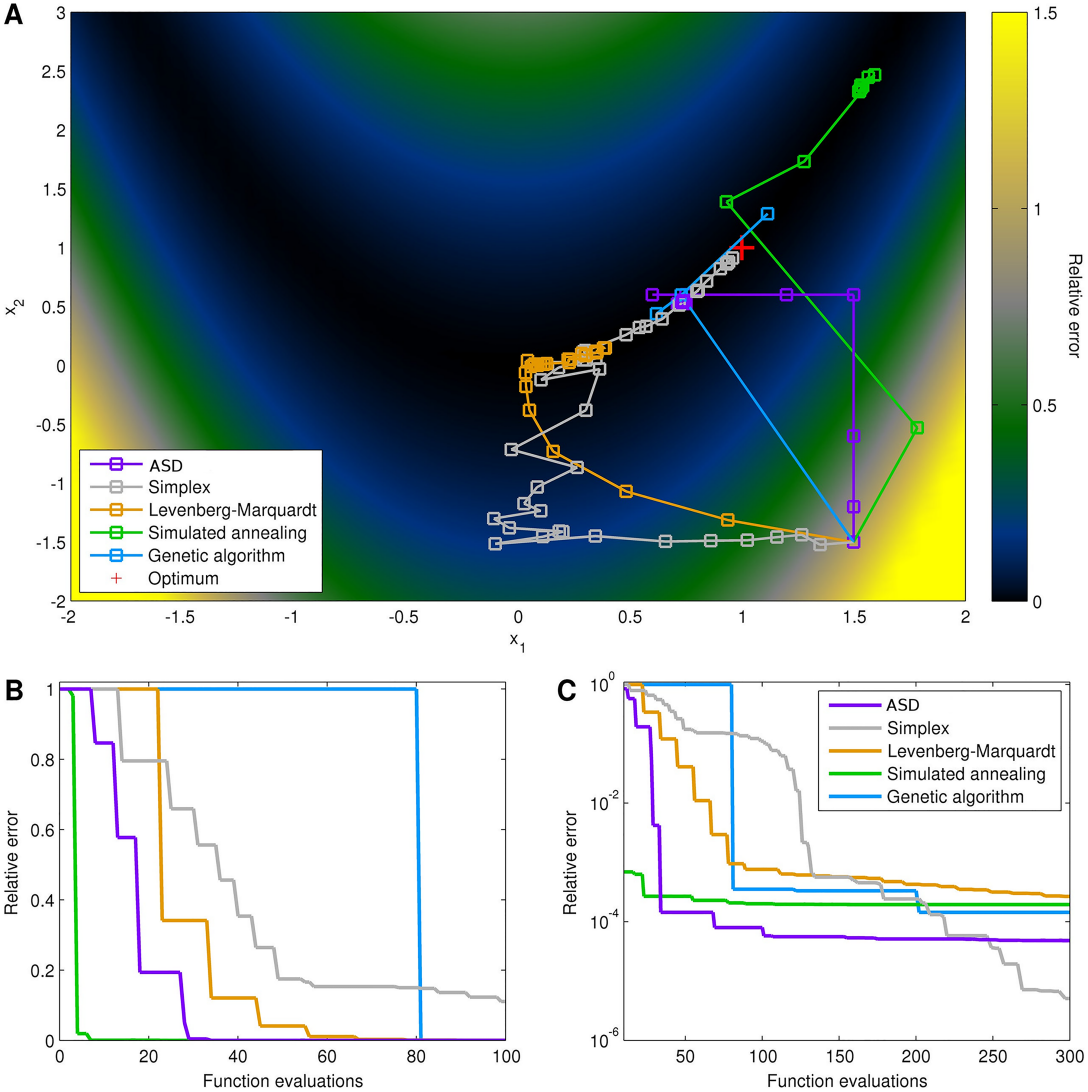
Optimization of the 10-dimensional version of Rosenbrock’s valley. (A) Trajectories of each optimization method starting up to 300 function evaluations from the starting point (1.5, −1.5); each iteration is shown with a square, but note that multiple function evaluations may occur at each iteration. Color shows error relative to starting point. Note the locally linear steps of ASD that rapidly adapt in size. (B) Relative error of each method for the first 100 function evaluations, showing the initial stage of the algorithms. (C) Relative error for the first 300 function evaluations, showing the asymptotic stage of the algorithms.

As shown in Fig 1, for the two-dimensional optimization problem, the nonlinear simplex method is most efficient, with all other algorithms requiring considerably more function evaluations to obtain the same error. Notably, after the initial descent, ASD was especially *inefficient*, since its assumption of local linearity is violated by the shallow, curved valley (if this assumption were relaxed, as described above, then ASD’s performance on this problem would be significantly improved). With the modified 10-dimensional version of Rosenbrock’s valley, ASD is the most efficient algorithm over most of the first several hundred function evaluations, as shown in Fig 2 for a single random seed. For small numbers of iterations (<30), for this particular seed, simulated annealing was by far the most efficient algorithm, reducing the error by a remarkable 98% after just 4 function evaluations. However, this algorithm became mired near the point (1.5, 2.4), far from the minimum of (1, 1), and did not significantly reduce the error beyond the first 20 function evaluations. After 50 function evaluations, ASD had reduced the error by a median of 99.9%, compared to 99.7% for simulated annealing, 96% for the Levenberg-Marquardt method, 82% for the nonlinear simplex method, and 0% for the genetic algorithm. Similarly, ASD reduced the error by 99.99% after 70 function evaluations; in comparison, the next best algorithm (the simplex method) required 220 function evaluations to reach the same error level.

During the descent into the shallow curved valley (comprising ∼99.9% of the total error), the most efficient algorithms were ASD and simulated annealing; within the valley (the remaining ∼0.1% of the total error), the simplex algorithm was by far the most efficient. Hence, these examples illustrate that in optimization problems where some parameters are significantly more important than others, ASD has significant advantages. In contrast, for problems in which all parameters have equal importance, as in the original Rosenbrock’s valley problem, other algorithms have superior performance.

For the 4-dimensional Powell’s quartic function, the nonlinear simplex method was again the most efficient, followed by ASD. For the 12- and 20-dimensional version, ASD was most efficient for 60–1700 and 250–4400 function evaluations respectively (corresponding to roughly 99.9999% of the total error at the upper limit in each case), after which the simplex method was most efficient. For the 100-dimensional version, the Levenberg-Marquardt method was most efficient for the first 1000 function evaluations (corresponding to 97% of the total error), but ASD was the most efficient algorithm for larger numbers of function evaluations. In practice, algorithms are not run for a fixed number of function evaluations, but rather until they satisfy a given stopping criterion, which is usually defined in terms of the change in the relative or absolute error. Specific choices for these criteria depend on the problem at hand, but for illustrative absolute error tolerances of 99.9% or 99.99%, ASD was the most or equal-most efficient for all cases except the 2D version of Rosenbrock’s valley.

**Fig 3:**
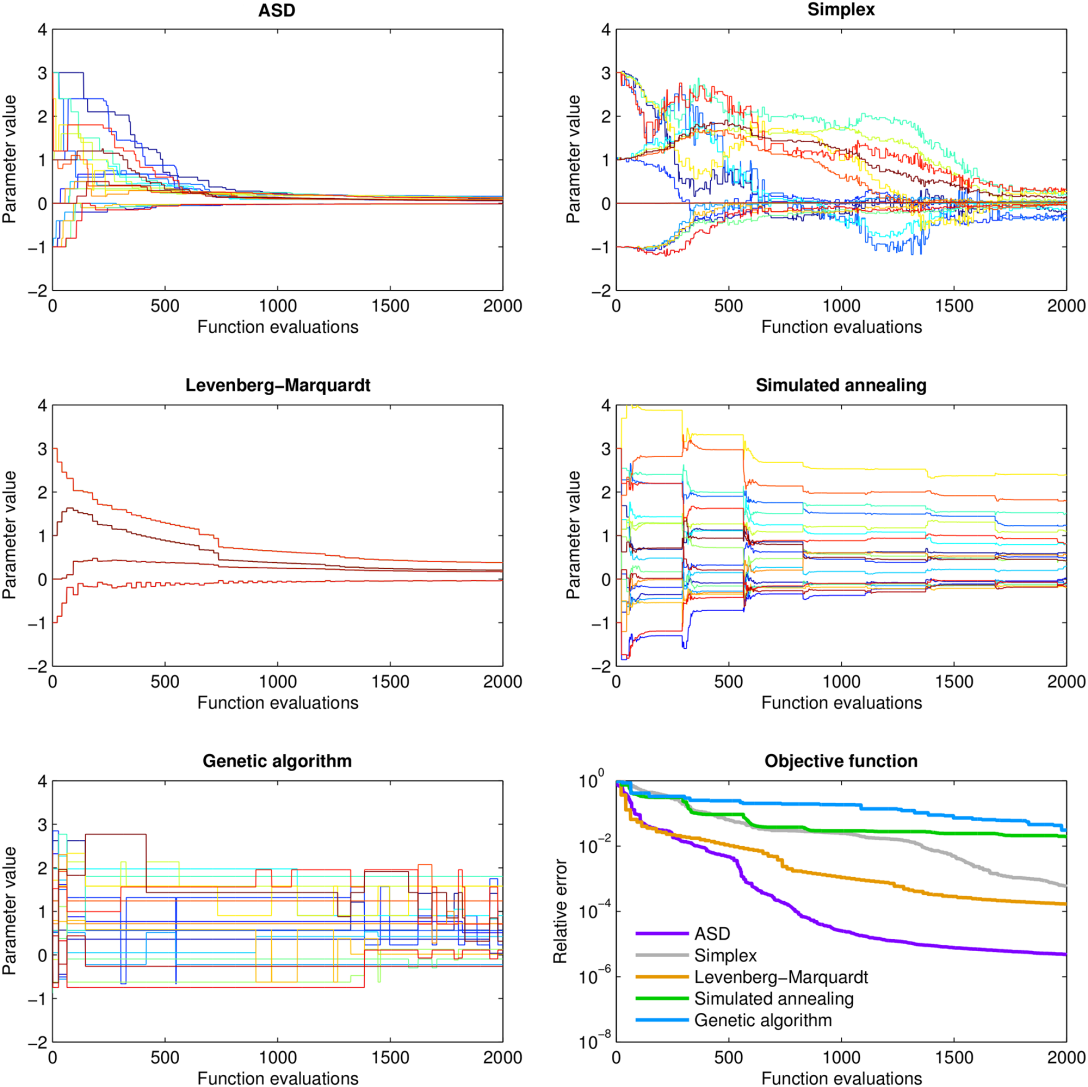
Demonstration of parameter update strategies for each algorithm applied to a 20-dimensional Powell’s quartic function. Each plot has 20 lines, showing the value of each parameter after each function evaluation. The optimum is at (0, 0, 0, … 0), corresponding to all 20 lines converging to 0. The error relative to the starting point for each method is shown in the bottom right panel. For small numbers of iterations (the adaptive phase of ASD), the Levenberg-Marquardt method reduces error most quickly; for larger numbers of iterations, ASD achieves 1–4 orders of magnitude smaller error for a given number of iterations than the other methods. (Note: since the genetic algorithm does not use a single initial point, individuals were instead initialized using a uniform random distribution in the range [−1, 3]. The Levenberg-Marquardt algorithm operates on the 20-dimension Powell’s function identically to the 4-dimensional version, with the exception that each iteration requires 5 times as many function evaluations.)

The five optimization methods discussed here employ very different parameter update strategies, as shown strikingly in Fig 3. The approach used in ASD is most similar to the Levenberg-Marquardt method, with the exception that the rate of convergence of the former *increases* over time (due to its adaptive step size), whereas for the latter, and for other algorithms, it decreases (as expected from Donsker’s theorem [35]). In the example shown here (a 20-dimensional Powell’s quartic function), the Levenberg-Marquardt method has the lowest error for 250 or fewer iterations; for large numbers of iterations, ASD has by far the lowest error—indeed, for 2000 or more iterations, it has nearly 2 orders of magnitude less error than the Levenberg-Marquardt method, and 4 orders of magnitude less error than nonlinear simplex, simulated annealing, and genetic algorithms. The superior performance of ASD compared to the other methods is surprising since, unlike in Fig 2, in this problem all parameters are of roughly equal importance, so the adaptive probability **p** is unlikely to significantly contribute to the efficiency of the optimization. Thus, even in cases where ASD’s only advantage is its adaptive step size, it is still capable of outperforming traditional algorithms.

## Optimizing HIV resource allocations

In contrast to the foregoing theoretical discussion of error minimization for analytical functions, here we describe the practical application that ASD was designed for: finding the allocation of resources across different HIV prevention and treatment programs that minimizes new infections [36]. To do this, we used the Optima HIV model (formerly known as Prevtool [15]) to perform the analyses. An overview of this version of the model is presented in S1 Appendix, with further details provided in [14]. Subsequent modifications to the model have been described in [37], and the most recent version of the software can be accessed via hiv.optimamodel.com.

In brief, the model describes HIV transmission and progression in a number of interacting subpopulations (14 in this case), including female sex workers, men who have sex with men, and general males and females in different age groups. The model incorporates parameters describing the sexual behavior, injecting behavior, HIV testing and treatment rates, and sexual and injecting partnerships of each population, as well as basic clinical parameters such as HIV transmissibility and disease progression rates. The model was based on to behavioral and surveillance data provided by the Swaziland Ministry of Health and UNAIDS. Further details are provided in [38]. In addition to empirical estimates of the model parameters, the model was calibrated to match surveillance data on HIV prevalence, diagnoses, and numbers of people on treatment. (Although ASD was also used for this calibration, here we instead focus on its use for the budget optimization procedure, since it better illustrates the differences between the methods.)

To optimize the allocation of Swaziland’s HIV budget, we assumed that spending on particular HIV programs produces changes in corresponding behavioral parameters or testing and treatment rates (for example, programs targeting female sex workers increase their probability of condom use). The objective being minimized was the number of new infections over the period 2015–2020, subject to the constraint that total funding was held constant for the last year in which full budget details were available (2014). The vector **x** being optimized consisted of the budget allocations across 9 different HIV prevention, testing, and treatment programs. Thus, the optimization problem had a dimensionality of 9 (since the constraint of constant total budget, which would otherwise reduce the dimensionality to 8, is applied post hoc). The initial budgets for different programs varied by over three orders of magnitude: from US$40,000 per year for prevention programs for men who have sex with men to US$45 million per year for antiretroviral treatment. To evaluate the objective function, the budget for each program was first converted to one or more model parameter values via a nonlinear cost-outcome function, which in turn were used in the nonlinear dynamical epidemic model. The cost-outcome functions and epidemic model are described in detail in S1 Appendix. Since the model is relatively computationally intensive, requiring approximately 1–2 s per function evaluation on a standard laptop, large numbers (>10^3^) of evaluations become wearisome.

This particular optimization problem has three notable aspects. First, despite the complexity and nonlinearity of the model, in almost all cases the objective function decreases monotonically as funding to any of the programs is increased—the only exception being HIV testing and counseling programs, in which case diagnosing more people with early-stage HIV infections without simultaneously increasing funding for antiretroviral treatment prevents some people with late-stage HIV infections from accessing treatment. Second, the country’s current HIV budget allocation, which is used as the initialization for the algorithm, is the product of considerable deliberation among numerous stakeholders and experts who have typically had the goal of allocating funds optimally. Thus, in most cases funds are already reasonably well allocated, and hence the initial starting point is expected to lie relatively close to the global optimum. Third, in situations where this is not the case, optimal solutions in very distant parts of parameter space are unlikely to be feasible given political and logistical constraints. Each of these three factors reduce the probability and/or importance of there being a difference between locally and globally optimal solutions.

**Fig 4:**
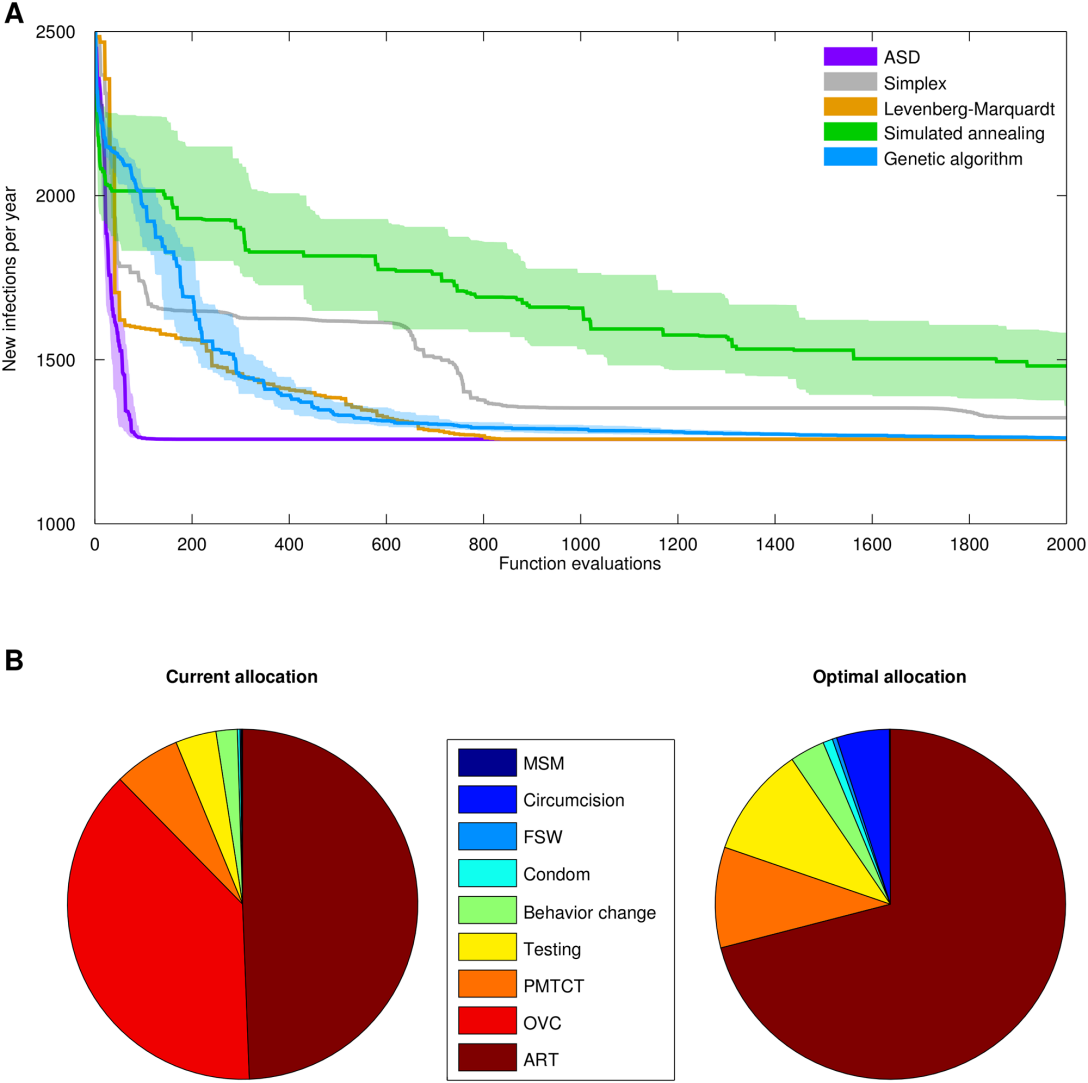
Comparison of optimization methods for a real-world example of HIV resource allocation. (A) Performance of each algorithm for the objective function (y-axis) of minimizing the number of new infections. As above, the shaded regions show the interquartile ranges over 40 different random seeds. (B) Original (left) and optimal (right) budgets. MSM = programs for men who have sex with men; Circumcision = voluntary medical male circumcision; FSW = programs for female sex workers; Comdom = condom promotion programs; Behavior change = social and behavior change communication; Testing = HIV testing and counseling services; PMTCT = prevention of mother-to-child transmission; OVC = programs for orphans and vulnerable children; ART = antiretroviral treatment.

As shown in Fig 4A, under current conditions, the model predicts approximately 2500 new infections per year in Swaziland. However, if funding is optimally allocated, as shown in Fig 4B (which consists largely of shifting funds from programs for orphans and vulnerable children towards treatment and male circumcision programs), this can be reduced to approximately 1260 new infections per year. ASD found this allocation after 65 function evaluations, while the next-best algorithm, the Levenberg-Marquardt method, found a nearly identical allocation after 830 function evaluations. None of the other methods reached this level of optimization within 2000 function evaluations; by that point, the genetic algorithm had achieved 99.3% of the reduction in new infections found by ASD and the Levenberg-Marquardt method, the nonlinear simplex algorithm 95%, and the simulated annealing algorithm 90%.

## Discussion and summary

This paper presents a simple optimization method inspired by the process of manual parameter fitting that is capable of outperforming traditional algorithms for certain classes of problems. The algorithm is most effective for problems with moderate to large dimensionality (≥5 dimensions), which corresponds to the case in which there are enough parameters that different parameters are likely to have substantially different overall contributions to the objective function. Indeed, the relative uniformity of parameters in the simple test functions used here (in terms of both scale and effectiveness) does not necessarily reflect certain real-world situations in which some—or even most—of the objective function’s parameters may have little influence on its value. In such situations, as with the real-world example of HIV budget allocations, ASD is especially effective, as it is able to adapt to those parameters (and those scales) that produce the greatest improvements in the objective function. An example of this is provided in Fig 4, where ASD finds what appears to be the globally optimal solution more than 10 times faster than any other algorithm. In contrast, ASD is less effective for optimization problems where the objective function has large discontinuties or numerous local minima; for such problems, evolutionary algorithms typically provide superior performance [39].

Within the taxonomy of optimization methods, ASD is a stochastic, derivative-free, direct search method (for an excellent review of random search methods for simulation optimization, see [40]). Thus, ASD is similar to adaptive random search algorithms [41–46]. However, these algorithms are adaptive only in terms of step size, not step probability, since typically they step in all dimensions simultaneously (*e.g.*, by sampling points from a hypersphere of radius equal to the current step size), and are thus unable to obtain information about individual dimensions. In addition, they typically require additional function evaluations to calculate the optimal step size, whereas ASD updates step size automatically on each iteration. ASD also has some similarities with tabu search [47], which updates step probability (by forming “taboos” about stepping in certain directions) but not step size. Thus, ASD is loosely analogous to a combination of the adaptive random search and tabu algorithms.

This study has two main limitations. First, we chose the four algorithms to compare against ASD based on their popularity, as evidenced by their inclusion in MATLAB’s Optimization Toolbox and Python’s SciPy module. However, as noted above, many other optimization algorithms exist, some of which significantly outperform these more traditional methods for particular problems—especially those that are non-convex, multi-modal, and/or have many local minima—as shown in the comprehensive review by Rios and Sahinidis [48]. Since ASD was intended as a relatively simple and general-purpose alternative to other traditional optimization algorithms, these more advanced algorithms and the complex (and often relatively specific) problems they have been designed to solve have not been considered in depth. The second limitation of this study is that MATLAB’s default values of the metaparameters were used for the simulated annealing and genetic algorithms (except the initial temperature of the simulated annealing, as noted above). Metaparameter tuning would likely increase the performance of these algorithms more than it would for ASD, since these algorithms are not adaptive—but conversely, an advantage of ASD is that it typically does not require any metaparameter tuning, so in that sense the comparison is fair. In this sense, ASD is highly unusual among random search methods in that it can be used “out of the box” with consistent performance across across a wide range of optimization problems for a default set of metaparameters; in contrast, metaparameter tuning is an essential step of using other methods [49].

As noted above, ASD has already been used successfully in the real-world applications of optimizing the allocation of HIV budgets, as well as calibrating various models—of HIV epidemiology, spiking neuronal network activity, and neural field dynamics—to experimental data. In the HIV budget optimization example shown above, standard optimization methods (including the four compared against ASD in this paper) were found to require an unpleasantly large number of function evaluations to obtain acceptable solutions. This led the authors to resort to manual parameter fitting until ASD was developed. It is our hope that this algorithm may be able to free other researchers from similar unpleasantries.

## Supporting information

S1 Appendix THIV epidemic model structure, methods, and data. (PDF)
