# Supplementary material for: Optimization by Adaptive Stochastic Descent
Source: PLoS One. 2018 Mar 16;13(3):e0192944. doi: 10.1371/journal.pone.0192944 (PMC5856269; doi:10.1371/journal.pone.0192944)
Supplement: S1 Appendix — (PDF) [file pone.0192944.s001.pdf]

Supplementary Methods

Contents

A. Model structure 2
B. Swaziland calibrations and cost-outcome relationships 5
B.1 Calibration 5
B.2 Cost-outcome relationships 6
C. Input data 7
C.1 Population size (thousands) 7
C.2 HIV prevalence (percent) 8
C.3 STI prevalence (percent) 8
C.4 Testing rate (%/year) 9
C.5 Treatment rate (%/year) 10
C.6 Mother-to-child transmission 10
C.7 Number of people on ART 10
C.8 Average number of regular sex acts (per person per year) 10
C.9 Average number of casual sex acts (per person per year) 11
C.10 Average number of commercial sex acts (per person per year) 12
C.11 Condom usage probability for regular acts (%) 12
C.12 Condom usage probability for casual acts (%) 13
C.13 Condom usage probability for commercial acts (%) 14
C.14 Circumcision probability (%) 14
C.15 Biological constants 16
C.16 Partnerships 18
C.17 Transitions 20
C.18 Economics 21

A. Model structure

The aim of the HIV transmission and progression model is to estimate the movement of people between population groups and between health states over time. Each combination of population group and health state is a single compartment in the model, and these evolve in time according to a set of differential equations. Seven types of movement are possible: people can enter the model by being born or leave the model by dying, or they can move between compartments by becoming infected, having their CD4 count decline, becoming diagnosed, or by going on or off treatment. Each compartment is described by a single ordinary differential equation, and each of the seven types of movement described above is a term in a differential equation. Positive terms result in an increase in population in a given compartment, while negative terms reduce the population of a given compartment. Movement between population groups (e.g., from female sex workers to low-risk females and vice versa; from male children to male adolescents, etc.) is also possible.

The model dynamics are determined by the initial conditions (i.e., the number of people in each compartment at the initial time point) and the model parameter values. Model parameters can depend on time t (e.g., 2002 vs. 2007), population group g (e.g., female sex workers vs. male injecting drug users), health state h (e.g. infected, CD4>500 vs. infected, CD4<200), and interaction type i (i.e., casual, regular, commercial, or injecting partnerships). The model supports up to 14 distinct population groups; since there are 21 health states (susceptible plus undiagnosed, diagnosed, first-line treatment, treatment failure, and second-line treatment for each of four CD4 stratifications), the model may have up to 294 compartments.

Transmission dynamics are determined by the rate at which susceptible (i.e., uninfected) individuals become infected. The probability that a susceptible individual in population group g_1 will be infected by an individual of population group g_2 at time t via sexual intercourse of interaction type i is defined as the force-of-infection:

$$\lambda(t, g_1, g_2, i) = 1 - \big(1 - \varsigma(t, g_1)\psi(t, g_1)I(t, g_2, i)\big)^{m(t, g_1, g_2, i)},$$

where λ is the force-of-infection; ς is the effect of circumcision; ψ is the effect of coinfection with ulcerative STIs; I is the infectiousness, which is determined by prevalence and transmissibility; and m is the number of unprotected acts.

The effect of circumcision is defined as $\varsigma(t, g) = 1 - e_\varsigma p_\varsigma(t, g)$, where e_ς is the fractional reduction in transmission probability per act due to circumcision (i.e., its efficacy), and p_ς is the probability that an individual is circumcised. This probability is zero for female populations, so circumcision only affects male populations. The effect of STI coinfection is $\psi(t, g) = e_\psi p_\psi(t, g)$, where e_ψ is the fractional increase in transmissibility caused by ulcerative STIs (such as HSV-2 and syphilis), and p_ψ is the probability of infection (i.e., prevalence) of these STIs.

The infectiousness of a population is

$$I(t, g) = \sum_h P(t, g, h)\beta(h),$$

where I is the infectiousness, β is the biological transmission probability, which depends on whether the intercourse is insertive, vaginal receptive, or anal receptive; P is the prevalence of each health state (such that $P(t, g) = \sum_h P(t, g, h)$ is the prevalence of HIV in that population); and $\beta(h)$ is the biological transmission probability, which depends on the viral load, which in turn can be approximated from the individual's CD4 count category h.

The number of unprotected acts is $m(t, g_1, g_2, i) = n(t, g_1, g_2, i)\big(1 - e_\kappa p_\kappa(t, g_1)\big)$, where n is the number of acts between individuals in two populations, e_κ is the fractional reduction in transmission probability per act due to condom use, and p_κ is the probability of condom use. While it may seem odd that the effect of condom use appears in the exponent while the effect of circumcision appears in the

base term, circumcision affects the biological transmission probability and thus affects the risk per act, whereas condom use can be considered as a reduction in the number of acts. The two approaches are equivalent to first order, and since the transmission probability is typically $<10^{-3}$, it makes negligible difference whether these terms appear in the base or exponent.

The force-of-infection due to injecting drug use is

$$\lambda_d(t) = 1 - \big(1 - \chi(t) I_d(t)\big)^{\omega(t) m_d(t)},$$

where λ_d is the force-of-infection for injection interactions between injecting drug users, χ is the effect of syringe cleaning, I_d is the infectiousness of injecting drug users, ω is the effect of methadone treatment, and m_d is the number of receptively shared injections.

The effect of syringe cleaning is $\chi(t) = 1 - e_\chi p_\chi(t)$, where e_χ is the fractional reduction in transmission probability per act, and p_χ is the probability that a syringe is cleaned before use. The infectiousness I_d has the same form as before, but now includes only injecting drug users. The effect of methadone is $\omega(t) = 1 - e_\omega p_\omega(t)$, where e_ω is the fractional reduction in the number of injections, and p_ω is the probability that a given injecting drug user is on methadone. The number of receptively-shared injections is given by $m_d(t) = n_d(t) s(t)$, where n_d is the total number of injections, and s is the probability that a given syringe has been receptively shared prior to use.

Finally, the force-of-infection for a given population group g_1 at a given point in time t is the product of all individual forces-of-infection (including injecting interactions, if applicable):

$$\Lambda(t, g_1) = 1 - \prod_{g_2} \prod_{i} \big(1 - \lambda(t, g_1, g_2, i)\big)$$

This quantity is the instantaneous risk of an individual in population group g_1 becoming infected. The indices g_2 and i include all interactions an individual has. For example, direct female sex workers have commercial interactions with male clients, as well as regular and casual relationships with all four heterosexual male populations, for a total of nine interaction types.

In contrast to the force-of-infection, most types of movement between population compartments are described by a single rate, and thus the remaining equations are relatively straightforward. Labeling the health states h such that 1=CD4>500, 2=350<CD4<500, 3=200<CD4<350, and 4=CD4<200, the full set of differential equations is as follows.

The change in the number of susceptible individuals is

$$\frac{dS(t,g)}{dt} = \varepsilon(t,g) - \big(\Lambda(t,g) + \mu_{0g}\big) S(t,g),$$

where S is the number of susceptible individuals, ϵ is the entry rate into the population, λ is the force-of-infection, and μ_{0g} is the background mortality rate for population group g. Note that the term ΛS in this equation is the number of new infections, while λS would be the incidence.

The change in the number of infected but undiagnosed individuals is

$$\frac{dU_{h=1}(t,g)}{dt} = \Lambda(t,g)\, S(t,g) - \big(\mu_{hg} + \zeta_h(t,g) + \pi_h\big) U_h(t,g),$$
$$\frac{dU_{h>1}(t,g)}{dt} = \pi_{h-1} U_{h-1}(t,g) - \big(\mu_{hg} + \zeta_h(t,g) + \pi_h\big) U_h(t,g),$$

where U_h is the number of undiagnosed individuals in health state h (note that newly infected individuals ΛS only enter $U_{h=1}$, the CD4>500 compartment), μ_{hg} is the mortality rate for the given health state and population group, ζ_h is the testing rate, and π_h is the disease progression rate ($\pi_{h=4} = 0$, since no progression occurs after CD4<200).

The change in the number of diagnosed individuals is

$$\frac{dD_h(t,g)}{dt} = \zeta_h(t,g)U_h(t,g) + \pi_{h-1}D_{h-1}(t,g) - \left(\mu_{hg} + \tau_h(t,g) + \pi_h\right)D_h(t,g),$$

where D_h is the number of infected and diagnosed individuals in a particular health state h, and τ_h is the HIV treatment rate.

The change in the number of individuals on treatment or with treatment failure is

$$\frac{dA_1(t,g)}{dt} = \sum_h \sigma_h(t,g)D_h(t,g) - (\mu_A + \phi_1)A_1(t,g),$$
$$\frac{dA_2(t,g)}{dt} = \sigma_F(t,g)A_F(t,g) - (\mu_A + \phi_2)A_2(t,g),$$
$$\frac{dA_F(t,g)}{dt} = \phi_1 A_1(t,g) + \phi_2 A_2(t,g) - (\mu_F + \sigma_F(t,g))A_F(t,g),$$

where A_1, A_2, and A_F are the numbers of people on first- and subsequent-lines of ART and treatment failure, respectively; μ_A and μ_F are the mortality rates for individuals on ART and with treatment failure, respectively; and ϕ_1 and ϕ_2 are the failure rates for first- and subsequent-lines of ART, respectively.

This set of coupled differential equations can be solved numerically; in practice, maximum computational efficiency is achieved by converting these to difference equations with a suitable time step (usually 0.1–1 years). Running the MATLAB implementation of this model for 10 population groups for a 50-year time span with an 0.2-year time step on an Intel Xeon 2.4 GHz CPU takes approximately 2 s.

B. Swaziland calibrations and cost-outcome relationships

This section describes the model calibration and cost-outcome relationships that were used to analyze the Swaziland epidemic and calculate the optimal resource allocations.

B.1 Calibration

By adjusting appropriate behavioral and clinical parameters, we manually calibrated the model to the HIV epidemic in Swaziland from 2000 to 2012 to match prevalence estimates for each population group, as well as the number of people on treatment. The resulting set of parameters and corresponding epidemic projection represents the baseline simulation. These calibrations are shown in **Figure S1**.

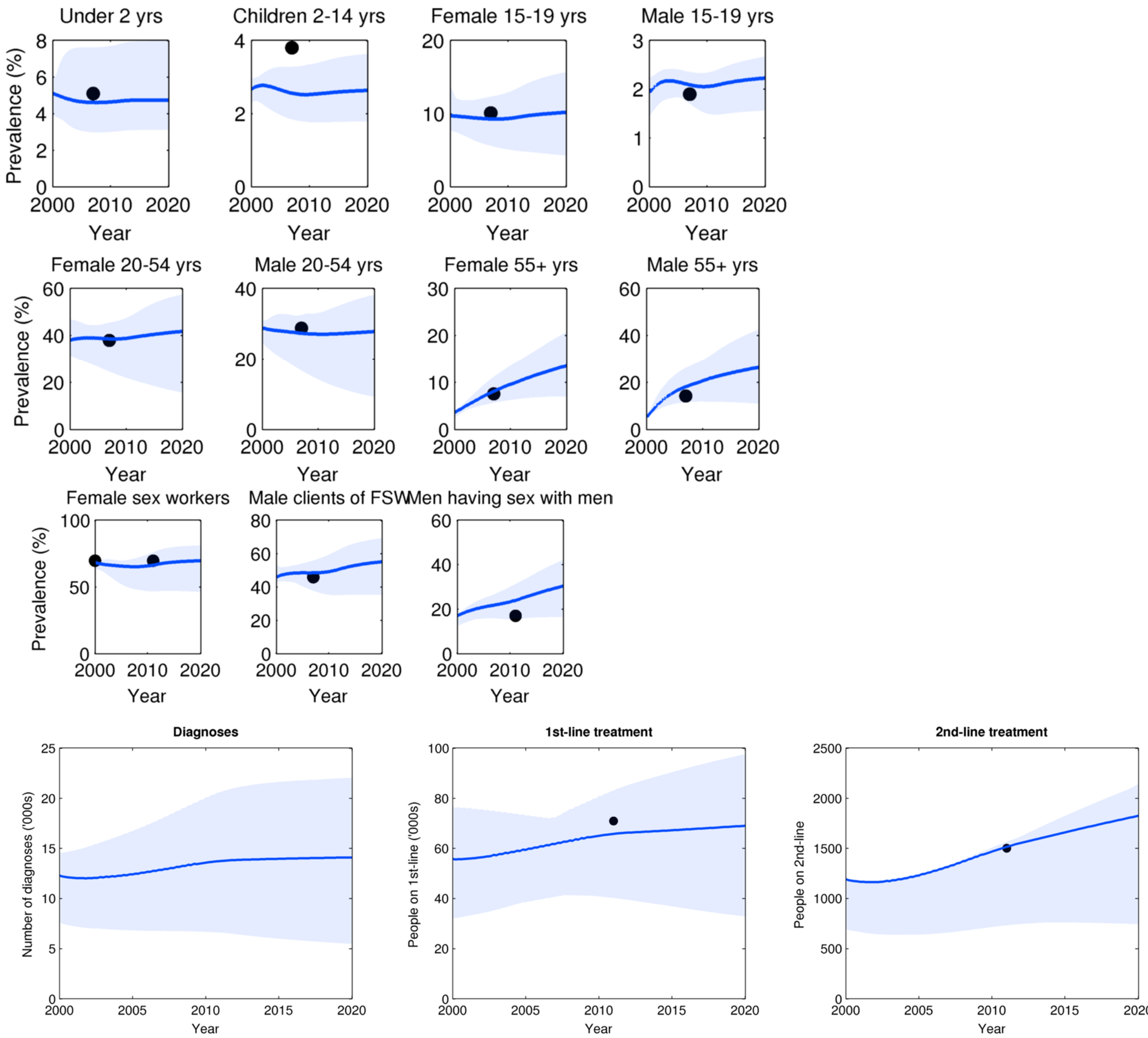

Figure S1: Calibration of the model to the HIV epidemic in Swaziland. Black discs represent available data for HIV prevalence, and the number of people on first- and second-line antiretroviral treatment. Solid curves are the best fitting simulation; shaded regions show uncertainty in the simulation.

B.2 Cost-outcome relationships

Resource optimization was performed by relating HIV program spending to changes in behavior via series of cost-outcome relationships. These relationships are shown in **Figure S2**.

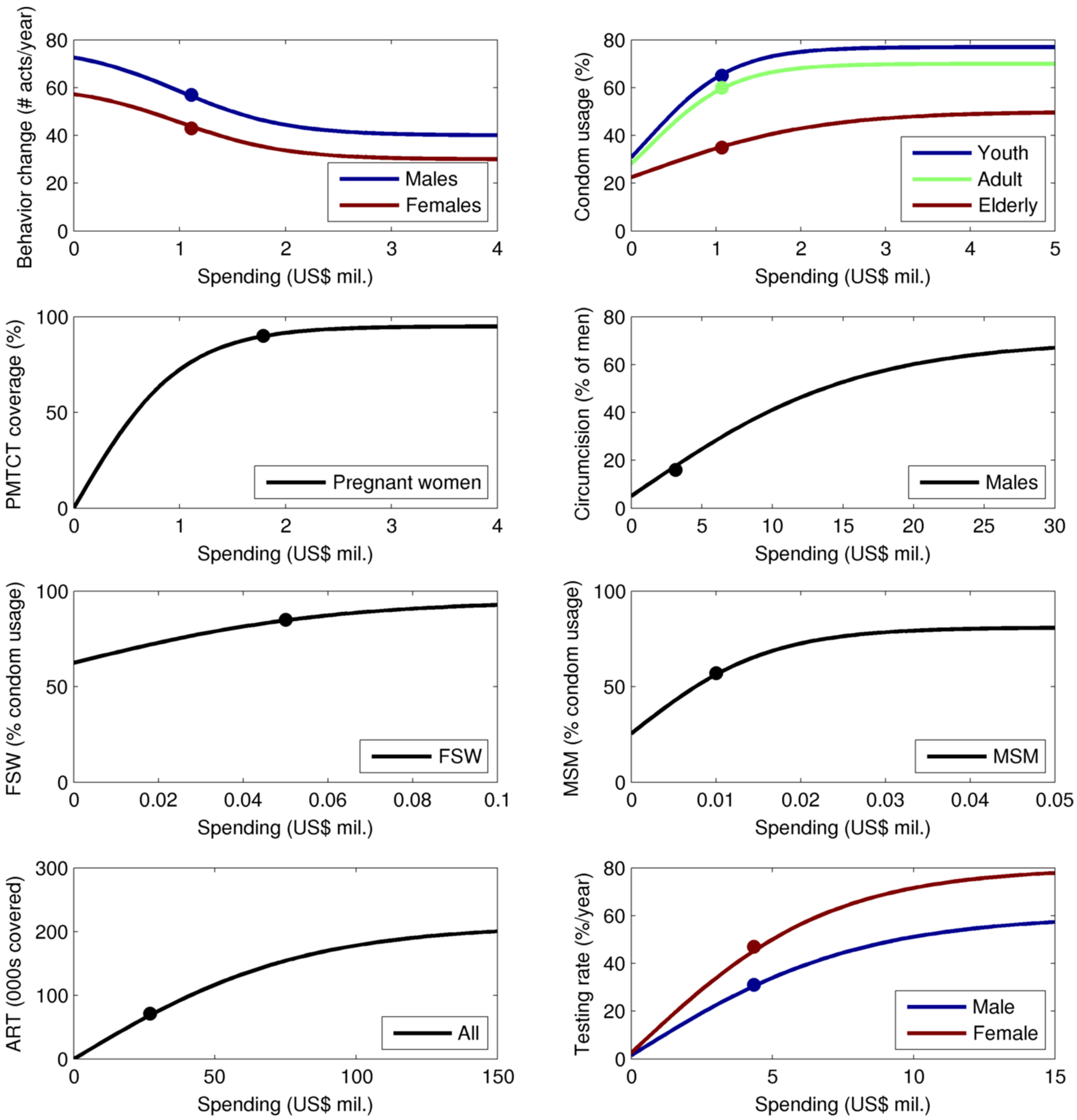

Figure S2: Logistic cost-outcome relationships for Swaziland. The spending axis is in millions of 2012 US dollars and is the overall spending allocated to each population for HIV prevention or treatment. Black discs represent available spending data and associated behaviors. The zero spending outcome data are based on the values available for the year 2000. The solid curve is the best fitting or assumed relationship used in our analysis.

C. Input data

These tables list all input data available for Swaziland. Assumptions are indicated by minus signs; no quantities are actually negative. Population group abbreviations are as follows:

INF: Infants (<2 years)
CHLD: Children (2-10)
FYTH: Females (10-14)
MYTH: Males (10-14)
FTEEN: Females (15-19)
MTEEN: Males (15-19)
FYAD: Females (20-24)
MYAD: Males (20-24)
FAD: Females (25-49)
MAD: Males (25-49)
FOLD: Females (>50)
MOLD: Males (>50)
FSW: Female sex workers
MSM: Men who have sex with men

C.1 Population size (thousands)

	2000	2001	2002	2003	2004	2005	2006	2007	2008	2009	2010	2011
INF	87											
CHLD	243											
FYTH	62											
MYTH	61											
FTEEN	53											
MTEEN	50											
FYAD	43											
MYAD	35											
FAD	126											
MAD	100											
FOLD	52											
MOLD	42											
FSW	3.04											
MSM	3.552											

C.2 HIV prevalence (percent)

	2000	2001	2002	2003	2004	2005	2006	2007	2008	2009	2010	2011
INF	-5.1							5.1				
CHLD	-3.8							3.8				
FYTH	-10.1							10.1				
MYTH	-3							1.9				
FTEEN	-16							16				14
MTEEN	-3							3				1
FYAD	-38							38				31
MYAD	-12							12				7
FAD	-40							40				48
MAD	-40							40				38
FOLD	-21							21				32
MOLD	-28							28				42
FSW	-69.7											69.7
MSM	-17.1											17.1

C.3 STI prevalence (percent)

	2000	2001	2002	2003	2004	2005	2006	2007	2008	2009	2010	2011
INF								0				
CHLD								0.05				
FYTH								2.4				
MYTH								1.04				
FTEEN								4				
MTEEN								3				

FYAD								6.93				
MYAD								6.49				
FAD								6.5				
MAD								1				
FOLD								1				
MOLD								1.3				
FSW								16.1				
MSM								15.8				

C.4 Testing rate (%/year)

	2000	2001	2002	2003	2004	2005	2006	2007	2008	2009	2010	2011
INF							5					
CHLD							0.5					
FYTH							10					
MYTH							1.8					
FTEEN							10					
MTEEN							1.8					
FYAD							26					
MYAD							12.3					
FAD							26					
MAD							12.3					
FOLD							10.2					
MOLD							9.8					
FSW											61.7	

MSM											51	
AIDS stage											80	

C.5 Treatment rate (%/year)

	2000	2001	2002	2003	2004	2005	2006	2007	2008	2009	2010	2011
CD4(500)								3.6				
CD4(350,500)								7.4				
CD4(200,350)								27.5				
CD4(200)								57.8				
Treatment failure								-1				

C.6 Mother-to-child transmission

	2000	2001	2002	2003	2004	2005	2006	2007	2008	2009	2010	2011
Birth rate (births/woman/year)			0.13									
Pregnant women covered (%)			95									

C.7 Number of people on ART

	2000	2001	2002	2003	2004	2005	2006	2007	2008	2009	2010	2011
1st-line												70901
2nd-line												1501

C.8 Average number of regular sex acts (per person per year)

	2000	2001	2002	2003	2004	2005	2006	2007	2008	2009	2010	2011
INF								0				
CHLD								0				
FYTH								10				
MYTH								1				
FTEEN								13.2				

MTEEN								5.23				
FYAD								43				
MYAD								20				
FAD								43				
MAD								40				
FOLD								4.7				
MOLD								32.1				
FSW												7.5
MSM												37.6

C.9 Average number of casual sex acts (per person per year)

	2000	2001	2002	2003	2004	2005	2006	2007	2008	2009	2010	2011
INF								0				
CHLD								0.16				
FYTH								15				
MYTH								2				
FTEEN								14.55				
MTEEN								7.22				
FYAD								15.4				
MYAD								26.5				
FAD								15.4				
MAD								10				
FOLD								0.6				
MOLD								3.6				

FSW												43.5
MSM												33

C.10 Average number of commercial sex acts (per person per year)

	2000	2001	2002	2003	2004	2005	2006	2007	2008	2009	2010	2011
INF												
CHLD												
FYTH												
MYTH												
FTEEN												
MTEEN												
FYAD												
MYAD								2				
FAD												
MAD								2				
FOLD												
MOLD								3				
FSW												228
MSM												10

C.11 Condom usage probability for regular acts (%)

	2000	2001	2002	2003	2004	2005	2006	2007	2008	2009	2010	2011
INF								0				
CHLD								52.5				
FYTH								35				

MYTH								45				
FTEEN								35				
MTEEN								45				
FYAD								27				
MYAD								36				
FAD								27				
MAD								36				
FOLD								15				
MOLD								20				
FSW												48.9
MSM												51.9

C.12 Condom usage probability for casual acts (%)

	2000	2001	2002	2003	2004	2005	2006	2007	2008	2009	2010	2011
INF												
CHLD								52.5				
FYTH								51.9			66.3	
MYTH								68.8			93.6	
FTEEN								51.9			66.3	
MTEEN								68.8			93.6	
FYAD								55.5				
MYAD								67.9				
FAD								55.5				
MAD								67.9				

FOLD								35				
MOLD								35.3				
FSW												48.9
MSM												57.1

C.13 Condom usage probability for commercial acts (%)

	2000	2001	2002	2003	2004	2005	2006	2007	2008	2009	2010	2011
INF												
CHLD												
FYTH												
MYTH												
FTEEN												
MTEEN												
FYAD												
MYAD								75				
FAD												
MAD								75				
FOLD												
MOLD								75				
FSW												85
MSM												57

C.14 Circumcision probability (%)

	2000	2001	2002	2003	2004	2005	2006	2007	2008	2009	2010	2011
INF								0.5				
CHLD								2				

FYTH												
MYTH								4.2				
FTEEN												
MTEEN								4.2				
FYAD												
MYAD								10.1				
FAD												
MAD								10.1				16
FOLD												
MOLD								11.9				
FSW												
MSM								10.1				

C.15 Biological constants

**Interaction-related transmissibility (% per act):**	Male & female (insertive)	0.09
	Male & female (receptive)	0.25
	Male & male (insertive)	0.02
	Male & male (receptive)	0.02
	Injecting	0.3
	Mother-to-child	-35

**Disease-related transmissibility**	CD4(500)	4
	CD4(350,500)	1
	CD4(200,350)	1
	CD4(200)	3.8
	Treatment	0.25

**Disease progression rate: (% per year)**	CD4 (500) to CD4 (350,500)	24.5
	CD4 (350,500) to CD4 (200,350)	51
	CD4 (200,350) to CD4 (200)	51

**Treatment recovery rate: (% per year)**	CD4 (350,500) to CD4 (500)	45
	CD4(200,350) to CD4 (350,500)	70
	CD4 (200) to CD4 (200,350)	36

**Death rate: (% mortality per year)**	Background	1.45
	Injecting	1
	CD4 (500)	0.0515

	CD4 (350,500)	0.128
	CD4 (200,350)	1.1
	CD4 (200)	50
	Treatment (CD4<200)	4

**Treatment failure rate: (% per year)**	1st-line	4.5
	2nd-line	4.5

**Efficacy/change in transmissibility due to:**	Condom (%)	80
	Circumcision (%)	60
	Diagnosis (%)	-1
	STI cofactor increase (%)	700
	Syringe cleaning (%)	75
	Methadone (%)	95
	PMTCT (%)	-78
	Treatment risk compensation (%)	-100

C.16 Partnerships

Entries indicate insertive populations (rows) and receptive populations (columns). Numbers indicate relative partnership formation probability; e.g., male teenagers are three times as likely to pair with female teenagers as they are with female youth, hence the entries of 1 and 3 in the corresponding cells of the table below.

Regular sexual interactions

	INF	CHLD	FYTH	MYTH	FTEEN	MTEEN	FYAD	MYAD	FAD	MAD	FOLD	MOLD	FSW	MSM
INF														
CHLD														
FYTH														
MYTH			1											
FTEEN														
MTEEN			1		3									
FYAD														
MYAD			1		3		9							
FAD														
MAD			1		3		9		9					
FOLD														
MOLD					1		3		9		9			
FSW														
MSM														1

Casual sexual interactions

	INF	CHLD	FYTH	MYTH	FTEEN	MTEEN	FYAD	MYAD	FAD	MAD	FOLD	MOLD	FSW	MSM
INF														
CHLD														
FYTH														
MYTH			1											
FTEEN														
MTEEN			1		3									
FYAD														
MYAD			1		3		9							
FAD														
MAD			1		3		9		9					
FOLD														
MOLD					1		3		9		9			
FSW														
MSM														1

Other sexual interactions

	INF	CHLD	FYTH	MYTH	FTEEN	MTEEN	FYAD	MYAD	FAD	MAD	FOLD	MOLD	FSW	MSM
INF														
CHLD														
FYTH														
MYTH														
FTEEN														
MTEEN														
FYAD														
MYAD													1	
FAD														
MAD													1	
FOLD														
MOLD													1	
FSW														
MSM														

C.17 Transitions

Optima allows two types of transition: in asymmetric transitions, individuals move from one population (rows) to another (columns), and individuals are not replaced. In symmetric transitions, the number of people leaving the first population is replaced by an equal number of people from the second population. For example, aging is an asymmetric transition, whereas it is assumed that female sex workers can transition into the low-risk population and vice versa, so this is a symmetric transition.

Asymmetric population transitions (% leaving per year)

	INF	CHLD	FYTH	MYTH	FTEEN	MTEEN	FYAD	MYAD	FAD	MAD	FOLD	MOLD	FSW	MSM	Total
INF		50													50
CHLD			6	6											12
FYTH					20										20
MYTH						20									20
FTEEN	-12.4						20								7.6
MTEEN								20							20
FYAD	-12.4								20						7.6
MYAD										20					20
FAD	-12.4										4				-8.4
MAD												4			4
FOLD															0
MOLD															0
FSW															0
MSM															0

Symmetric population transitions (% leaving per year)

	INF	CHLD	FYTH	MYTH	FTEEN	MTEEN	FYAD	MYAD	FAD	MAD	FOLD	MOLD	FSW	MSM	Total
INF															0
CHLD															0
FYTH															0
MYTH															0
FTEEN															0
MTEEN															0
FYAD															0
MYAD															0
FAD															0
MAD															0
FOLD															0
MOLD															0
FSW							5		5						10
MSM															0

C.18 Economics

		2000	2001	2002	2003	2004	2005	2006	2007	2008	2009
Health-care costs (US$/year)	CD4>500 (per person per year)										566.6
	500>CD4>350										213.9
	350>CD4>200										348.6
	CD4<200										426.5
	HIV testing (per test)										7.2
	1st-line ART										519
	Subsequent ART										1176.7

		Value
Health utilities (QALYs)	Uninfected IDUs	0.86
	Untreated HIV, CD4>500	0.72
	Untreated HIV, 350<CD4<500	0.57
	Untreated HIV, 200<CD4<350	0.43
	Untreated HIV, CD4<200	0.14
	Treated HIV, CD4>500	0.762
	Treated HIV, 350<CD4<500	0.756
	Treated HIV, 200<CD4<350	0.716
	Treated HIV, CD4<200	0.645
